# A Targeted and an Untargeted Metabolomics Approach to the Volatile Aroma Profile of Young ‘Maraština’ Wines

**DOI:** 10.3390/metabo12121295

**Published:** 2022-12-19

**Authors:** Ana Boban, Urska Vrhovsek, Silvia Carlin, Ana Mucalo, Irena Budić-Leto

**Affiliations:** 1 Institute for Adriatic Crops and Karst Reclamation, 21 000 Split, Croatia; 2 Department of Food Quality and Nutrition, Edmund Mach Foundation, Research and Innovation Centre, 38010 San Michele all’Adige, Italy

**Keywords:** aroma, volatile compounds, two-dimensional gas chromatography, Maraština wines, spontaneous fermentations

## Abstract

This study investigated the detailed volatile aroma profile of young white wines of Maraština, *Vitis Vinifera* L., produced by spontaneous fermentation. The wines were produced from 10 vineyards located in two Dalmatian subregions (Northern Dalmatia and Central and Southern Dalmatia). Volatile compounds from the wine samples were isolated by solid-phase extraction (SPE) and analyzed by an untargeted approach using two-dimensional gas chromatography coupled with time-of-flight mass spectrometry (GC×GC/TOF-MS) and a targeted approach by gas chromatography–tandem mass spectrometry (GC-MS/MS). A comprehensive two-dimensional GC×GC analysis detailed the total volatile metabolites in the wines due to its excellent separation ability. More than 900 compounds were detected after untargeted profiling; 188 of them were identified or tentatively identified. A total of 56 volatile compounds were identified and quantified using GC-MS/MS analysis. The predominant classes in Maraština wines were acids, esters, and alcohols. The key odorants with odor activity values higher than one were *β*-damascenone, ethyl caprylate, ethyl isovalerate, ethyl 2-methylbutyrate, ethyl caproate, isopentyl acetate, ethyl butyrate, and phenylacetaldehyde. The metabolomics approach can provide a large amount of information and can help to anticipate variation in wines or change winemaking procedures.

## 1. Introduction

Aroma is one of the most important quality attributes of wine, and the perceived flavor is the result of complex interactions between all the volatile and nonvolatile compounds. The aroma of young wine consists of compounds derived from grapes and those produced from alcoholic fermentation [1]. Traditional winemaking practices rely on the microbiota naturally present in the grapes and in the winery environment. Yeasts belonging to the genera *Saccharomyces cerevisiae* will eventually dominate and complete fermentation, but it takes time to establish the fermentation. During this time, many other indigenous yeast genera belonging to the non-*Saccharomyces* species have a greater role in flavor development than *S. cerevisiae* through extracellular enzymes. They can liberate glycosidically bound constituents and contribute significantly to the character and quality of the final wine [2]. There is a growing interest in native microflora towards possible contribution to the aroma features linked to *terroir* influences and the expression of these attributes. Nowadays, authors have pointed out that the presence of natural microbiota in wine fermentation that relies on wine regions significantly contributes to the specific flavor characteristics of wine. Wines made from the same grape variety but from different geographical locations are appreciated for their diversity [3,4]. Recently, 25 different fungal genera present in Maraština grapes have been characterized in the indigenous microbiota of Maraština grapes collected from vineyards located within the Croatian coastal wine-growing region of Dalmatia (Northern Dalmatia, Dalmatian hinterland, and Central and Southern Dalmatia) [4].

Many different methods for studying wine aroma have been developed using a targeted approach, especially one-dimensional gas chromatography (1D-GC) coupled with different detectors [5]. One-dimensional gas chromatography coupled with tandem mass spectrometry (1D-GC-MS/MS) is one of the most efficient analytical techniques for metabolomics studies [6]. Due to the rapid development of analytical chemistry within the last decades encompassing a tandem mass detector, the determination of the exact concentrations of compounds present in trace amounts is a challenge. A triple quadrupole detector mass spectrometer (QqQ-MS) operating in a selected reaction monitoring mode is the advanced method in more detailed quantitative metabolomics studies. Besides its sensitivity, this instrument has a very good linear dynamic range, which allows excellent quantification of the metabolites of different chemical classes in a five-fold and even higher concentration range [7]. However, this targeted approach does not provide full information about volatile components. The 1D-GC volatile fractions are hampered by frequent co-elution, even when high-efficiency capillary columns, selective stationary phases, and programmed oven temperature conditions are used. An untargeted metabolomics approach by comprehensive two-dimensional gas chromatography coupled with a time-of-flight mass spectrometer (GC×GC/TOF-MS) emerged as a more powerful analytical technique for the detailed analysis of the volatile compounds of complex samples such as wines [8]. This technique utilizes a long non-polar column with a short polar column connected by a modulator. The instrument’s heart is the modulator because it ensures that separation is comprehensive and multidimensional. GC×GC allows the separation of a large number of compounds in a single chromatographic run due to the added selectivity of the second column and inherently high peak capacity [9]. Using this instrumental approach, compounds co-eluting from the first column undergo additional separation in the second one [10]. Therefore, the separation potential, with higher peak capacity, selectivity is greatly enhanced when compared to the one-dimensional GC. 

Maraština, a Croatian autochthonous variety of grape, is one of the most important white cultivars in the Adriatic coastal region of Croatia and has the potential for producing high-quality monovarietal and dessert wines [11,12]. Maraština wines are characterized by a higher intensity of yellow color and distinguished from Chardonnay, Istrian Malvasia, and Muscat blanc wines by the more intense body, viscosity, astringency, and tannin quantity [13]. Maraština wines produced from different vine-growing subregions in Dalmatia have indicated significantly different basic physico-chemical parameters of the must and color intensities of wine [14]. According to the legislation, Croatian wines produced from different viticultural areas of Dalmatia can be labeled with a protected designation of origin (PDO) (Regulation EU, No. 1308/2013) [15].

In this study, we thoroughly examined the volatile aroma profile in experimental young Maraština wines produced by spontaneous fermentation in two vine-growing subregions of Dalmatia (the Northern Dalmatia subregion and the Central and Southern Dalmatia subregions) located along the Adriatic coast. This study aimed to discriminate the wines produced in those two subregions based on volatile aroma profiling. To date, information on the volatile composition of Maraština wines produced from spontaneous fermentation has not been found, so this investigation fulfills the knowledge of Croatian wines. Profiling by comprehensive GC×GC/TOF-MS was combined with a conventional GC-MS/MS analysis of volatile compounds to obtain wine volatile metabolome. 

## 2. Methods and Materials

### 2.1. Chemicals and Reagents

Ethanol 99.8%, n-heptanol 99.9%, dichloromethane 99.8%, and methanol for HPLC 99.9% were purchased from Sigma-Aldrich (Sigma-Aldrich, St. Luis, MO, USA). Milli-Q water was used for the extraction of samples and the preparation of standard solutions. Cartridges with 200 mg of stationary phase based on styrene–divinylbenzene for solid-phase extraction (SPE) were purchased from Isolute^®^ ENV+ (Biotage, Uppsala, Sweden).

### 2.2. Vineyard Parcel Characteristics

The Maraština vineyards were selected to represent the major soil and climate types of the two Dalmatian subregions. The five commercial vineyards and the germplasm collection at the Institute for Adriatic Crops in the Central and Southern subregions (CSD) and four commercial vineyards in the Northern Dalmatia (ND) subregion were chosen for the production of experimental wines. Table S1 summarizes the main characteristics of the vineyard parcels under study, such as soil type, plantation year, altitude, row distance, and row orientation. Row orientation in the vineyards was north–south in both subregions. The vineyards in the CSD subregion were situated on reddish-brown soil on limestone with a sandy loam texture at an altitude from 14 to 94 m above sea level (a.s.l.). The three vineyards in the ND subregion were situated on brown soil on limestone with a sandy texture, whereas one vineyard was on reclaimed karst. The vineyards were at altitudes from 60 to 260 m a.s.l. All the vines were trained to the vertical shoot-positioned bilateral cordon system and were cultivated without irrigation. The canopy management techniques were the same in both subregions, all vines were pruned to four cuttings with two buds, and thinning was performed when the shoots were 15 cm long.

### 2.3. Wine Samples

A total of 30 wines made from the Maraština variety were produced by spontaneous fermentation, without added inoculated yeast. In each vineyard, nine representative vines were chosen randomly within the vineyard during the 2021 vintage. The grapes were harvested in technological maturity separately determined for each subregion due to the different climatic conditions. From each vineyard, 15 kg of grapes were harvested and stored in a cooler during transport to Institute for Adriatic Crops. The grapes were destemmed, crushed, and treated with potassium metabisulfite to give a total concentration of SO_2_ in the wine of approximately 50 mg/L. The must was separated and cold-stabilized for 24 h at 4 °C. The stabilized must from each repetition was decanted in 500 mL Erlenmeyer flasks and protected from light with aluminum foil. The fermentations were carried out at 20 °C. The fermentation progress was monitored daily by measuring the sugar content and fermentation temperature. Samples of the young wines from the end of the fermentation were taken in 50 mL falcon tubes and stored at −80 °C until the metabolomics analysis of aroma.

### 2.4. Climate Data

The climate in Northern Dalmatia subregion and Central and Southern Dalmatia subregions is Mediterranean, based on climate data from the meteorological station (Vela Luka, Split, Zadar, and Benkovac). The average temperature in the period from January 2021 to September 2021 was 16.8 °C for ND and 18.0 °C for CSD subregions. June was the driest month with an average precipitation of 3 mm (ND) and 5 mm (CSD). Most of the precipitations during the growing season occurred in April (50 mm in ND) and May (66 mm in CSD). More detailed climatological data for the year 2021 are reported in Table S2.

### 2.5. Solid-Phase Extraction for GC-MS/MS and GC×GC/TOF-MS Analysis

Sample preparation and extraction were performed according to the modification of the previously described method [16]. Isolute^®^ ENV+ solid-phase extraction cartridges were supplied by Biotag (Uppsala, Sweden) filled with 200 mg of stationary phase. The cartridge was pre-conditioned with 4 mL of dichloromethane, followed by 4 mL of methanol and 4 mL of model wine solution. A total of 50 mL of wine mixed with 100 µL of internal standard (n-heptanol, 250 mg/L) was added to the cartridge, washed with 3 mL of Milli-Q water, and dried for 10 min. The extracted compounds were eluted directly into the injection vial from the cartridge with 2 mL of dichloromethane.

### 2.6. GC-MS/MS Analysis

Analysis was performed using the Agilent Intuvo 9000 system for fast GC coupled with an Agilent 7010B triple quadrupole mass spectrometer (QqQ) (Agilent Technologies, Santa Clara, CA, USA) equipped with an electronic ionization source operating at 70 eV. Separation was obtained by injecting 1 µL in split mode (1:10) into a DB-Wax Ultra Inert column (30 m × 0.25 mm id × 0.25 µm film thickness, Agilent Technology, Santa Clara, CA, USA). The initial temperature of the GC oven was 40 °C for 2 min, increased by 10 °C/min to reach 55 °C, then by 20 °C/min until 165 °C, by 40 °C/min to 240 °C for 1.5 min, and, finally, by 50 °C/min to 250 °C and kept at this temperature for an additional 4 min (16 total runtimes). Helium was used as a carrier gas (with a flow of 1.2 mL/min). The mass spectra were acquired in multiple reaction monitoring modes. Nitrogen was used as the collision gas, with a flow of 1.5 mL/min, in addition to Helium at 4.0 mL/min as a quench gas. The transfer line and source temperature were set at 250 °C and 230 °C, respectively. The data acquisition and subsequent analyses were performed using the MassHunterWorkstation software 10.0.368 (Agilent Technologies, Santa Clara, CA, USA) [16].

### 2.7. GC×GC/TOF-MS Analysis

The GC×GC system consisted of an Agilent 7890N (Agilent Technologies, Palo Alto, CA, USA) coupled with a LECO Pegasus IV time-of-flight mass spectrometer (TOF-MS) (Leco Corporation, St. Joseph, MI, USA) equipped with a Gerstel MPS autosampler (GERSTEL GmbH & Co. KG, Mülheim an der Ruhr, Germany), as described in previous studies with modifications [17]. A volume of 1 µL of wine extract (SPE) was injected at 250 °C in split mode (1:10). The oven was equipped with a 30 m × 0.25 mm × 0.25 µm film thickness VF-WAXms column (Agilent Technologies, Santa Clara, CA, USA) in the first dimension (1D) and a 1.5 m × 0.15 mm × 0.15 µm film thickness Rxi 17Sil MS column (Restek, Bellefonte, PA, USA) in the second dimension (2D). The primary oven temperature was kept at 40 °C for 4 min, then raised at 6 °C/min to 250 °C, and then finally maintained at this temperature for an additional 5 min. The secondary oven was maintained at 5 °C above the temperature of the primary oven throughout the chromatographic run. As described previously [18], the modulator was offset by +15 °C in relation to the secondary oven; the modulation time was 7 s with 1.4 s of hot pulse duration. Helium was used as a carrier gas at a constant flow of 1.2 mL/min. The MS parameters included electron ionization at 70 eV, with ion source temperature at 230 °C, a detector voltage of 1317 V, a mass range of 40–350 *m*/*z*, an acquisition rate of 200 spectra/s, and an acquisition delay of 120 s. Automated peak finds and spectral deconvolution with a baseline offset of 0.8 and a signal-to-noise (S/N) ratio of 100 were performed using LECO ChromaTOF software version 4.32 (Leco Corporation, St. Joseph, MI, USA). Peak width limits were set to 42 s and 0.1 s in the first and the second dimension, respectively. Adaptive integration was not used. The required match (similarity) to combine peaks was set to 650. Under these conditions, 938 putative compounds were detected. Volatile compounds were identified by comparing their retention times and mass spectra with those of pure standards and with mass spectra from NIST 2.0, Wiley 8, and FFNSC 2 (Chromaleont, Messina, Italy). Mass spectrometric information of each peak was compared to NIST mass spectra libraries, with a minimum library similarity match of 750. A mix of 122 compounds was injected under identical conditions to identify compounds by comparison with pure standards. Tentative identification of wine aroma compounds and/or confirmation of their identities was achieved by comparing experimental linear temperature-programmed retention index (LTPRI) with those from the literature for conventional one-dimensional GC obtained using columns of equal or equivalent polarity (NIST 2.0, Wiley 8, FFNSC 2, VCF).

### 2.8. Data Analysis

The statistical analyses of the volatile compounds were carried out by using IBM^®^SPSS^®^ Statistica for Windows program package version 23.0 (SPSS Inc., Chicago, IL, USA). Statistically significant differences between mean values at *p* < 0.05 were obtained by one-way ANOVA and the least significant difference (LSD) test. Multivariate analyses were performed on reduced data sets. The Fisher F-ratio was used for the selection of the parameters. The initial GC-MS data set of 56 volatile compounds was reduced to 15 variables. This reduced data set was used for principal component analysis. Additionally, the initial data set of 188 volatile compounds determined by GC×GC/TOF-MS was reduced to 56 compounds for performing hierarchical clustering. Heatmap was generated by Ward algorithm and Euclidean distance analysis using the metabolomics data analysis program MetaboAnalyst v.5.0. (http://www.metaboanalyst.ca) (accessed on 5 November 2022) created at the University of Alberta, Canada [19].

## 3. Results and Discussion

The wine subregion according to the legislation of the Republic of Croatia (Regulation NN 32/2019) has been proposed as a marker for the production of wine with a protected designation of origin. The vine-growing subregion represents a geographically limited area with similar climatic and pedological conditions and other agrobiological conditions, which enable the production of wine with the specific characteristic of the subregion. The results of this study were obtained from experimental wines belonging to the two vine-growing subregions: Northern Dalmatia (ND) and Central and Southern Dalmatia (CSD). In the current study, the vineyards of the ND subregion were located at higher altitudes, mainly situated on brown soil on limestone. The vineyards of the CSD subregion, located in the central and southern parts of Dalmatia, were planted on reddish-brown soil. Regarding the temperature data, vineyards in ND were exposed to a 1.3 °C lower average temperature and lower average precipitation during the vegetation period. Numerous studies show that soil type, climate, training systems, canopy, and cultural practices strongly impact the shoot growth, yield per vine, and the aroma composition of the berries [11,17,20]. 

### 3.1. GC-MS/MS Analysis

The concentrations of all quantified volatile aroma compounds in young Maraština wines by targeted approach with the GC-MS/MS method are presented in Table 1. The compounds are sorted by chemical classes and descending Fisher *F*-ratio in each group. A total of 56 volatile compounds were quantified, including terpenic compounds (14), C_13_-norisoprenoids (3), esters (17), alcohols (4), acids (5), phenols (4), aldehydes (2), ketones (2), lactones (4), and indole (1).

In Maraština wines, 15 volatile compounds were significantly different among the two vine-growing subregions in Dalmatia. The obtained results are in agreement with previous studies on Australian wines from different wine-growing regions, which showed the influence of climate conditions on alternations of volatile precursors, which can modify the fermentation medium and lead to changes in the aroma profile of wine [17]. It was shown that compounds associated with wines from the cooler climate were grape-derived volatiles, such as monoterpenes, C_6_ compounds, and some C_13_-norisoprenoids [17]. The higher rainfall promotes a decrease in the concentration of volatiles [21]. 

Terpenic compounds were the largest group of primary aroma compounds identified in the wines of Maraština. In this research, the two vine-growing subregions were significantly different in the concentration of *trans*-linalool oxide, *cis*-linalool oxide, *cis*-rose oxide, and *trans*-rose oxide (*p* < 0.05). *β*-citronellol (10.01 µg/L), linalool (6.88 µg/L), geraniol (5.23 µg/L), and *α*-terpineol (1.71 µg/L) were determined in the highest concentration in young Maraština wines derived from the ND subregion. Additionally, similar concentrations were determined in the CSD region, which shows a match with previous studies on white wines [22,23]. Linalool has characteristic citrus-like, sweet, and flowery notes; *β*-citronellol, *α*-terpineol, and geraniol exhibit flowery and sweet aromas [24]. In this study, all identified terpenic compounds were present in concentrations lower than their sensory threshold. Still, with a relatively wide array of present fruity–sweet–citric–flowery notes, there is a synergistic contribution to wine aroma [25]. Luzzini and co-workers reported a higher concentration of *trans*-linalool and *cis*-linalool oxide in spontaneous fermentation [26]. Rose oxide is a typical compound in Traminette wine with a lychee aroma [27], but it was not identified in wines produced from spontaneous fermentation [26]. The Gewürztraminer wine, with concentrations of linalool, *α*-terpineol, and rose oxide, which are similar in concentrations to our results, was described with notes of tropical fruit and ginger aromas [28]. Among the compounds that are related to discrimination with grape varieties, terpenic compounds were found to be highly discriminant and, thus, confirm the fact as being good markers of origin [13,29]. Additionally, it has been observed that concentrations of terpenic compounds were impacted by different yeasts in alcoholic fermentation [30].

C_13_-noriseprenoids are the second group of compounds belonging to the varietal aroma. Grapes accumulate a wide range of C_13_-noriseprenoids whose aglycones contribute highly desirable flavor and aroma properties [31]. In Maraština wines from both vine-growing subregions, *β*-damascenone, 1,1,6-trimethyl-1,2-dihydronaphthalene (TDN), and vitispirani (mix of isomers) were detected. The concentration of *β*-damascenone (1.89 µg/L in ND) in Maraština is above the odor perception threshold, 0.05 µg/L [32] (Table S3). *β*-damascenone had a direct impact on wine aroma with an odor reminiscent of honey, prunes, or overmatured plums. In the ND subregion, significantly higher concentrations of TDN and *β*-damascenone were detected. This observation is consistent with Loyd and co-workers [33], who emphasize the importance of grape growing conditions in relation to concentrations of *β*-damascenone. TDN has been highlighted as a compound whose concentration increases for grapes grown under higher sunlight exposure, which is related to the ND subregion [34]. 

Esters contribute to the fruity and floral characteristics and aroma complexity of wines, even at concentrations below their odor threshold, by synergistic effect [35]. Through fatty acid acyl- and acetyl coenzyme A (CoA) pathways, yeasts produce ethyl fatty acid esters during alcoholic fermentation. On the other hand, acetate esters are produced through the condensation of higher alcohols with acetyl-CoA, which are under the control of esterase enzymes [36]. The most abundant ester in this study was isoamyl acetate, with concentrations of 491.93 µg/L in the ND subregion and 579.53 µg/L in the CSD subregion, followed by ethyl caprylate. Those esters contribute to the fresh fruity aromas of young white wines by commonly surpassing their low odor threshold, such as 30 µg/L for isoamyl acetate and 2 µg/L for ethyl caprylate [37]. The average concentration of ethyl isovalerate, ethyl 2-methylbutyrate, ethyl caproate, and ethyl butyrate surpassed their corresponding odor thresholds [38] (Table S3) and defined the fruity–flowery component of the aroma profile of Maraština. Furthermore, the significantly higher total concentration of esters in wines from the ND subregion compared to the CSD subregion can be related to the colder climate and higher concentrations of fatty acids in the ND subregion [39]. The concentration of ethyl acetate and acetate esters increased in spontaneous fermentation compared to different *S. cerevisiae* strain-inoculated fermentations of Corvina and Corvinone wines [26]. Additionally, Canonico and co-workers [40] reported a positive effect of spontaneous fermentation on Verdicchio wine by producing the highest content of isoamyl acetate (653 µg/L).

An important part of the compounds derived from grape metabolism is C_6_ alcohols. Three of them, *cis*-hexen-1-ol, *trans*-3-hexen-1-ol, and 1-hexanol were quantified in Maraština. The most abundant C_6_ alcohol was 1-hexanol (303.70 µg/L in CSD), which could be related to the grape origin giving the vegetal character of wine [11]. C_6_ alcohol rarely directly participates in wine aroma due to a high odor perception threshold, such as 2500 µg/L for 1-hexanol [41]. The total concentrations of C_6_ alcohols were similar in both subregions (385.30 µg/L in the CSD subregion and 393.53 µg/L in the ND subregion). Some other studies on Corvina [26] and Chardonnay wines [42] showed that wines produced from spontaneous fermentation had lower concentrations of alcohols than other co-fermentations. 

Quantitatively, fatty acids were the larger group of secondary aroma compounds, followed by esters and alcohols. The total concentrations of fatty acids were significantly different between the two subregions. The major medium-chain fatty acids (MCFAs) quantified in Maraština wines are octanoic (2453.81 µg/L in ND), decanoic (967.72 µg/L in ND), and nonanoic (20.18 µg/L in CSD) acids. The concentration of octanoic acid was higher than their corresponding odor threshold of 500 µg/L [43] and significantly higher in the ND subregion. This trend was already observed by Petronilho and co-workers, who characterized the volatile fraction of the white wines Arinto and Bical and showed that fatty acids contribute to a large part of the aroma profile [44]. Yeasts are the primary producers of these fatty acids, which are worth mentioning because of their ability to convert to ethyl ester [45]. The different grape microbiotas of the wine subregions in Dalmatia described by Milanović [4] might influence the significant statistical difference in the acid content of young wine Maraština. Medina and co-workers reported elevated concentrations of MCFA during spontaneous fermentation in Chardonnay [42]. Inoculation with non-*Saccharomyces* and *Saccharomyces cerevisiae* can modify the chemical profile and bring benefits to regulating the content of fatty acids since their presence may have a negative impact on aromas with greasy and cheesy notes [46]. 

Volatile phenols are considered a characteristic compound in wine, but their influence on the final aroma can be positive or negative depending on their concentration. The main volatile phenols in wines are 4-ethylguaiacol, 4-vinylguaiacol, and 4-vinylphenol, which were all identified and quantified in examined Maraština wines too [41]. Volatile phenols can be produced from phenolic acids by yeast enzymatic activity or acid hydrolyses of their glycosides. The concentration of 4-vinylguaiacol in all investigated wines from CSD and ND subregions was higher than the odor percipient threshold of 40 µg/L [47], which is connected to negative clove notes. The presence of these compounds in wine is associated with *Brettanomyces* yeasts present in native microbiota [48]. 

Aldehydes and ketones are highly volatile constituents formed from yeasts during fermentation by decarboxylation of 2-oxo-3-phenylpropanoic acid or a chemical oxidation process [49]. Phenylacetaldehyde was significantly different in the Maraština wines from both subregions, and its concentrations were about 10 times higher than the sensory threshold of 4 µg/L [50]. Phenylacetaldehyde with OAV > 10 highly contributed to wine aroma with the key odorant of honey showing a significantly higher concentration in wines from ND. Similar data were found in other studies of wines where concentrations of phenylacetaldehyde were above the corresponding threshold, especially in young white wines [11,51].

Lactones are volatile organic compounds derived from lipid metabolism in grapes [52] and are naturally present in wine, especially *γ*-lactones and *δ*-lactones. These compounds had low perception thresholds (*γ*-nonalactone 25 µg/L and *γ*-octalactone 7 µg/L) [53,54] and very powerful odor descriptors that range from peach-like and coconutty to creamy and floral. Allamy reported concentrations of *γ*-nonalactone were low in white wines (about 5.9 μg/L), similar to our concentrations of 2.85 µg/L in ND and 2.27 µg/L in CSD [55]. Benzothiazole was the only indole detected in this study with similar concentrations in the ND (1.04 µg/L) and CSD (0.73 µg/L) subregions.

### 3.2. GC×GC/TOF-MS

Table 2 presents the volatile compounds that were identified or tentatively identified through a comparison of the experimental literature retention indices (LRI_exp_) and mass spectral data with corresponding data reported in the NIST database (LRI_lit_). A total of one hundred and eighty-eight identified or tentatively identified compounds included terpenic compounds (7), C_13_-norisoprenoids (1), esters (48), alcohols (25), acids (36), phenols (5), aldehydes (5), ketones (6), lactones and furanoids (22), sulfur-containing compounds (9), nitrogen-containing compounds (12), and other compounds (12). Compounds are listed according to different chemical classes and in order of decreasing *F*-ratio. It is evident that there are a large number of compounds that are co-eluted in the first dimension and which obviously cannot be properly observed with 1D-GC-MS. The use of GC×GC analyses resulted in 188 tentatively identified metabolites, a number that is three times higher than the one obtained by 1D-GC-MS. GC×GC/TOF-MS provides much-increased separation capacity and chemical selectivity for the analysis of metabolites present in a complex wine matrix. Wine metabolites are expressed as peak area and area percentage in two vine-growing subregions (CSD and ND) with their respective retention times in the first (^1^ tR) and in the second (^2^ tR) chromatographic dimensions, literature retention indices (LRI_lit_), and experimental retention indices (LRI_exp_) obtained in GC×GC/MS analyses.

Furthermore, a quantitative analysis would be necessary for a precise definition of the impact of volatile metabolites on wine aroma. Aroma descriptors found in the literature are employed for a general discussion regarding the influence of the presence of a volatile compound on the wine aroma. A discussion regarding the potential contribution of a few important metabolites is presented as follows. Terpenic compounds, including 8-hidroxylinalool, 2,3-dihydrofarnesol, hotrienol, *β*-citronellol, *trans*-farnesol, linalool, and geraniol were identified. The only identified C_13_-norisoprenoid was 3-oxo-*α*-ionol (0.04%) with a very similar chromatographic area in both subregions. These compounds have an impact on the aroma with notes that are floral with a slight woody note and notes of flowers, rose, and geranium. The number of detected terpenic compounds and C_13_-norisoprenoids was higher using the targeted approach—GC-MS/MS—because it is more sensitive and allows the quantification of terpenic compounds and norisoprenoids, even at very low concentrations.

The abundant classes were acids and esters with peak areas of 64.90% (CSD) and 66.63% (ND) showing correspondence with the results of GC-MS analysis. The compound with higher area percentages was hexanoic acid (7.32%). Additionally, the peak area of hexanoic acid showed significant differences in the two subregions, as well as 2-oxopentanedioic acid, acetic acid, isovaleric acid, butanoic acid, caprylic acid, octanoic, and succinic acid. Esters represented one of the most dominant classes of compounds, which is in the agreement with studies provided by GC×GC/TOF-MS [56,57], especially in the ND subregion. The higher areas of esters in Maraština wines belong to ethyl hydrogen succinate (6.44%), followed by ethyl 4-hydroxybutanoate (6.38%), diethyl butanedioate (4.84%), 2-methylbutyl acetate (4.78%), and ethyl 2-hydroxypropanoate (2.33%). Among the alcohols, the three major ones were: 2-phenylethanol (8.99%), butane-2,3-diol (6.23%), and heptan-1-ol (2.84%). The 2-phenylethanol, for example, contributes a positive rose aroma, and its presence was observed in the aroma of Merlot [57]. The next more abundant group was lactones and furanoids. 5-(hydroxymethyl) dihydrofuran-2(3H)-one (2.97%) and *γ*-butyrolactone (3.66%) had higher areas. *γ*-butyrolactone has sensory descriptors such as creamy and oily. The volatile sulfur compounds in wines come mainly from the metabolism of yeast and contribute mainly to unpleasant aromas in wines. Significantly different sulfur-containing compounds in the CSD and ND subregions were ethyl 3-methylthiopropanoate and 3-(methythio)propionic acid. The most abundant was 3-methylmercapto-1-propanol (1.98%). Moreira reported high levels of S-methyl thioacetate, 3-mercapto-1-propanol, 3-(ethylthio)-1-propanol, and 3-methylthiopropionic acid in white wines such as Alvarinho, Loureiro, and Avesso [58]. Out of a total of five phenols, 4-vinylguaiacol had the highest chromatography areas (0.56%). Nitrogen in wine is sourced from the degradation of amino acids and is used by yeast for the production of other nitrogen compounds. The most abundant nitrogen metabolites in the CSD and ND subregions were 2-ethylbutan-1-amine (0.19%), followed by N-phenethylacetamide, which were not identified by GC-MS. Among carbonyl compounds, 4-hydroxybenzaldehyde (0.14%) was found as a major chromatographic peak. Rodríguez-Bencomo and co-workers [59] reported this compound as one of the useful precursors that showed contents in grapes comparable to the levels observed in wine volatile compounds. The most important ketone was acetovanillone (0.10%). Acetovanillone is a component that is formed during wine oxidation [60].

### 3.3. Multivariate Statistical Analysis

**Figure 1:**
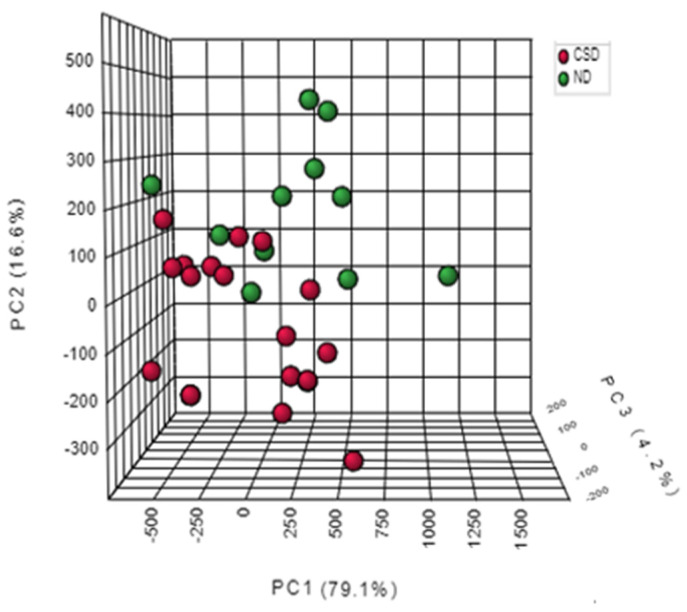
Separation of Maraština wines according to ND and CSD vine-growing subregions in three-dimensional space defined by the first three principal components PC1, PC2, and PC3.

The principal component analysis performed on the GC-MS data set allowed a good separation of Maraština wines derived from two vine-growing subregions. In a projection of 15 volatile compounds that defined the principal components PC1 and PC2, the first two principal components explained 95.7% of the variability (Figure 1). PC1 accounted for 79.1% of total variability, while PC2 accounts for 16.6% variability. Wines from the ND subregion were clearly differentiated from the wines from the CSD subregion along the direction of PC1 and gravitated to higher positive PC2 values.

**Figure 2:**
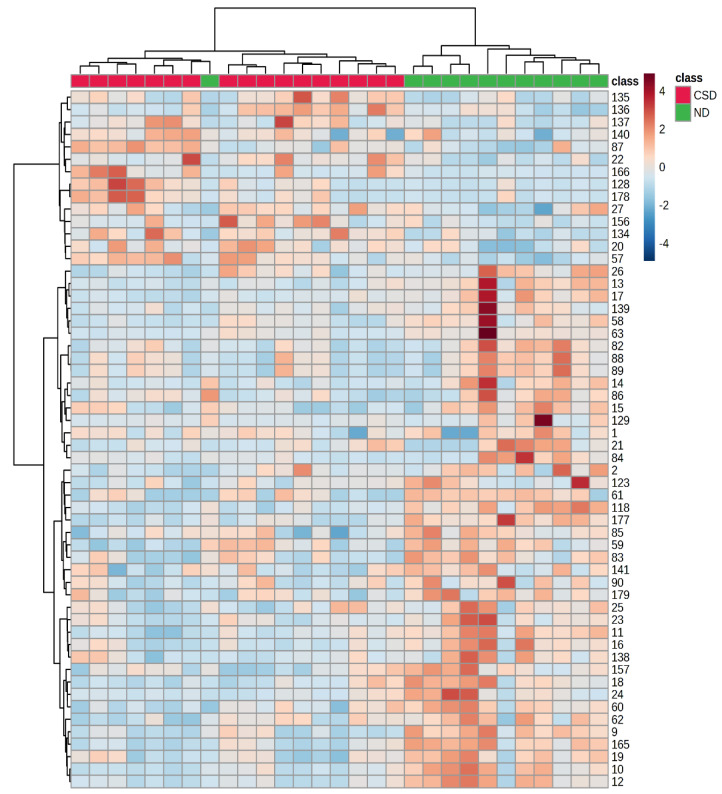
Hierarchical clustering representation corresponding to the 56 most significant volatile compounds of the Maraština wines from the two subregions (ND and CSD) obtained by GC×GC/TOF-MS analysis. The rows in the heat map represent compounds, and the columns indicate samples. Compounds are designated by numbers that correspond to those in Table 2. The relative content of each compound is illustrated through a chromatic scale (from dark-blue, minimum, to dark-red, maximum).

Hierarchical clustering analysis performed on the GC×GC/TOF-MS data set confirmed the discrimination of the Maraština wine volatile profile among the vine-growing subregions (Figure 2). On the heatmap, for the CSD subregion, a darker color in the column was evident for 10 compounds: δ-valerolactone, DL mevalolactone, 2,3-dihydro-1-benzofuran, hydroxy-4,4-dimethyldihydrofuran-2(3H)-one, acetic acid, methyl 4-hydroxybutanoate, methyl 2-methyl-3-oxobutanoate, ethyl pyruvate, 2,4,7,9-tetramethyl-5-decyne-4,7-diol, N-phenethylacetamide, 2-methyl-4-phenyl-3-pentanone, 1,1-di(2-methyl butoxy)ethane, ethyl 3-methylthiopropanoate, and 2-benzofuran-1(3H)-one. The rest of the 46 compounds had a higher chromatographic peak area in the ND subregion and mostly belong to terpenic compounds, esters, alcohols, and acids. Compounds with a darker color, which correspond to the higher chromatographic peak area, were 8-hydroxylinalool, hotrienol, decyl 2,2-dimethylpropanoate, 2-phenylethyl propionate, 3-methylpentan-1-ol, 4- methylpentan-1-ol, 4-hydroxybenzaldehyde, 4-hydroxy-6-pentyltetrahydro-2H-pyran-2-one, and 3-hydroxy-2-butanone.

## 4. Conclusions

In conclusion, young Maraština wines produced from the Northern Dalmatia subregion had a higher concentration of total volatile compounds than the Southern and Central Dalmatia subregions, especially the following compounds: *cis*-rose oxide, *trans*-rose oxide, *β*-damascenone, TDN, ethyl leucite, diethyl succinate, phenylacetaldehyde, benzaldehyde, and octanoic acid. The aroma profile of all experimental wines was dominated by esters, followed by acids and alcohols. Furthermore, the low odor thresholds and higher concentrations of compounds such as *β*-damascenone, ethyl caprylate, ethyl isovalerate, ethyl 2-methylbutyrate, ethyl caproate, isopentyl acetate, ethyl butyrate, and phenylacetaldehyde directly contribute to the aroma of young Maraština wines from both subregions with key odorants of fruity (apple, banana, strawberry, prune, and lemon) and honey notes. Spontaneous fermentations were characterized by the high concentration of esters regardless of grape origin and reflected in the distinctive aroma character of the wines. The methodology applied proved successful for the most detailed screening of metabolites in young Maraština wines produced by spontaneous fermentation reported to date. Different metabolomics approaches in this study (targeted and untargeted) made it possible to identify one hundred and eighty-eight compounds by GC×GC/TOF-MS and fifty-six compounds by GC-MS/MS. The metabolomics approach can provide a large amount of information and can help to anticipate variation in wines or change winemaking procedures. Multivariate analysis proved good separation and discrimination of Maraština wines from two Dalmatian subregions. 

## Figures and Tables

**Table 1:** Concentration (µg/L) of volatile aroma compounds (mean ± standard deviation) in young Maraština wines from Northern Dalmatia (ND) and Central and Southern Dalmatia (CSD) subregions determined by GC-MS/MS.

No.	Compound	t_R_ (min:s)	LOQ (µg/L)	*F*-Ratio	Concentration (µg/L)		S
					ND	CSD	
1	*cis*-Rose oxide	07:38.3	0.036	15.954	0.07 ± 0.03	0.04 ± 0.01	*
2	*trans*-Rose oxide	07:46.0	0.014	13.046	0.02 ± 0.01	0.01 ± 0.01	*
3	*cis*-Linalool oxide	08:29.9	0.114	10.211	0.27 ± 0.07	0.34 ± 0.06	*
4	*trans*-Linalool oxide	08:18.2	0.082	4.889	0.46 ± 0.11	0.54 ± 0.10	*
5	*trans*-Terpin	11:23.9	0.100	2.490	0.30 ± 0.14	0.23 ± 0.10	ns
6	1,8-Cineole	06:24.8	0.050	2.292	0.09 ± 0.04	0.07 ± 0.01	ns
7	α-Terpineol	09:51.2	0.100	1.634	1.71 ± 0.43	1.50 ± 0.45	ns
8	Eugenol	11:38.8	0.150	1.626	0.17 ± 0.07	0.22 ± 0.12	ns
9	Geraniol	10:28.8	0.250	1.062	5.23 ± 1.14	4.74 ± 1.35	ns
10	Terpinen-4-ol	09:21.5	0.075	0.475	0.13 ± 0.09	0.22 ± 0.44	ns
11	*β*-Ionone	10:55.2	0.050	0.340	0.07 ± 0.01	0.06 ± 0.03	ns
12	*β*-Citronellol	10:07.7	1.000	0.233	10.01 ± 3.79	9.45 ± 2.57	ns
13	Linalool	08:55.3	0.100	0.153	6.88 ± 1.23	6.63 ± 1.95	ns
14	Safranal	09:38.6	0.100	0.009	0.12 ± 0.06	0.12 ± 0.05	ns
	∑ Terpenic compounds				25.51 ± 3.84	24.18 ± 5.16	ns
15	*β*-Damascenone	10:27.8	0.100	38.577	1.89 ± 0.64	0.84 ± 0.27	*
16	TDN	10:09.0	0.050	16.381	0.68 ± 0.16	0.45 ± 0.15	*
17	Vitispirani (mix of isomers)	08:56.5	0.500	4.149	0.64 ± 0.31	0.39 ± 0.35	ns
	∑ C_13-_norisoprenoids				3.21 ± 0.76	1.67 ± 0.51	*
18	Ethyl caprylate	08:12.4	1.000	33.678	221.09 ± 68.24	176.73 ± 65.92	ns
19	Diethyl succinate	09:42.4	0.250	19.764	399.80 ± 195.07	170.80 ± 82.73	*
20	Ethyl valerate	05:37.9	0.050	4.846	1.22 ± 0.37	1.52 ± 0.35	*
21	Ethyl laurate	10:29.2	0.075	3.699	28.64 ± 46.24	7.47 ± 7.29	ns
22	Ethyl heptanoate	07:27.1	0.050	3.170	1.03 ± 0.24	1.42 ± 0.75	ns
23	Ethyl caprate	09:32.9	0.050	3.170	73.77 ± 73.89	45.76 ± 24.78	ns
24	Ethyl isovalerate	04:53.6	0.100	3.147	11.06 ± 6.23	8.02 ± 3.15	ns
25	Ethyl 2-methylbutyrate	04:42.5	0.050	3.097	7.55 ± 4.18	5.39 ± 2.56	ns
26	Ethyl leucate	08:55.9	0.250	2.878	36.78 ± 11.11	14.24 ± 9.94	*
27	Butyl acetate	04:55.8	0.150	1.549	0.59 ± 0.38	0.76 ± 0.38	ns
28	Ethyl caproate	06:36.0	0.050	1.532	178.75 ± 23.78	161.34 ± 44.5	ns
29	Isoamyl acetate	05:29.6	0.250	1.166	491.93 ± 309.43	579.53 ± 126.75	ns
30	Ethyl phenylacetate	10:16.3	0.050	1.067	4.73 ± 1.46	4.13 ± 1.62	ns
31	Isobutyl acetate	04:13.2	0.500	0.501	11.24 ± 6.80	12.49 ± 2.70	ns
32	Hexyl acetate	06:56.6	0.075	0.127	1.93 ± 2.62	2.24 ± 2.04	ns
33	Phenylethyl acetate	10:24.5	0.075	0.051	113.91 ± 30.45	110.87 ± 39.36	ns
34	Ethyl butyrate	04:30.0	0.100	0.001	67.10 ± 16.39	66.89 ± 15.94	ns
	∑ Esters				1651.12 ± 437.54	1369.58 ± 291.48	*
35	Benzyl alcohol	10:37.4	0.150	4.216	11.06 ± 3.39	16.07 ± 7.94	*
36	*cis*-3-Hexen-1-ol	07:49.3	0.014	1.664	52.28 ± 32.29	40.97 ± 15.4	ns
37	*trans*-3-Hexen-1-ol	07:39.6	0.050	0.590	28.76 ± 18.30	24.57 ± 11.70	ns
38	1-Hexanol	07:34.8	0.075	0.005	301.42 ± 75.5	303.70 ± 98.49	ns
	∑ Alcohols				393.53 ± 117.99	385.30 ± 115.34	ns
39	Geranic acid	12:13.3	5.000	8.813	4.32 ± 4.56	8.22 ± 2.65	*
40	Octanoic acid	11:14.2	50.000	5.916	2453.81 ± 420.15	2083.9 ± 400.08	*
41	Decanoic acid	11:57.5	50.000	4.083	967.72 ± 337.98	766.39 ± 209.3	ns
42	Nonanoic acid	11:35.3	10.000	1.833	18.18 ± 4.73	20.18 ± 3.36	ns
43	Valeric acid	09:59.8	5.000	0.903	41.03 ± 9.07	43.7 ± 6.34	ns
	∑ Acids				3480.74 ± 669.79	2914.16 ± 575.91	*
44	4-Vinylguaiacol	11:44.5	5.000	5.536	155.17 ± 107.71	271.54 ± 146.66	*
45	4-Ethyl phenol	11:37.7	0.050	4.260	0.08 ± 0.05	0.13 ± 0.06	*
46	Guaiacol	10:34.0	0.100	0.501	0.07 ± 0.07	0.09 ± 0.05	ns
47	4-Ethyl guaiacol	11:10.9	0.075	0.304	0.09 ± 0.03	0.10 ± 0.03	ns
	∑ Phenols				155.41 ± 107.82	271.85 ± 146.72	*
48	Phenylacetaldehyde	09:35.0	1.000	5.401	39.96 ± 6.79	31.85 ± 10.70	*
49	Benzaldehyde	08:51.4	0.150	1.056	0.21 ± 0.26	0.31 ± 0.27	ns
	∑ Aldehydes				40.16 ± 6.89	32.15 ± 10.78	*
50	2-Aminoacetophenone	11:52.7	0.050	1.017	0.21 ± 0.06	0.24 ± 0.08	ns
51	Zingerone	14:34.6	0.050	0.610	2.92 ± 1.81	3.42 ± 1.65	ns
	∑ Ketones				3.13 ± 1.79	3.66 ± 1.66	ns
52	*γ*-nonalactone	11:14.2	0.150	0.989	2.85 ± 2.11	2.27 ± 1.12	ns
53	*γ*-octalactone	10:50.6	0.100	0.568	1.90 ± 1.42	2.46 ± 2.28	ns
54	*γ*-decalactone	11:50.1	0.100	0.529	0.75 ± 0.15	0.80 ± 0.25	ns
55	*δ*-decalactone	11:38.0	0.150	0.009	8.61 ± 2.29	8.69 ± 2.26	ns
	∑ Lactones				14.11 ± 3.70	14.22 ± 3.86	ns
56	Benzothiazole	11:00.6	0.500	2.937	1.04 ± 0.76	0.73 ± 0.08	ns
	∑ Indole				1.04 ± 0.76	0.73 ± 0.08	ns

t_R_—retention time; LOQ—limit of quantification; S—statistical differences; ns—no significant differences; and * —significant differences (*p* < 0.05). *Cis* and *trans* indicate geometric isomers and are written in italic type.

**Table 2:** Chromatographic area and area percentage (%) of volatile aroma compounds in young Maraština wines from Northern Dalmatia (ND) and Central and Southern Dalmatia (CSD) subregions determined by GC×GC/TOF-MS, sorted by compound class, and in descending Fisher *F-*ratio.

No.	Compound	*m*/*z*	1 tR (min:s)	2 tR (min:s)	LRIexp	LRIlit	ND		CSD		F	S
							Area	%	Area	%		
1	8-Hydroxylinalool	101	30:42.0	00:01.2	2300	2294	6426	0.01	5214	0.01	5.608	*
2	Hotrienol	71	18:48.0	00:01.3	1604	1605	4887	0.00	2844	0.00	5.067	*
3	2,3-Dihydrofarnesol	69	30:07.0	00:01.6	2273	2265	18,585	0.02	15,003	0.02	1.936	ns
4	*β*-Citronellol	69	21:50.0	00:01.4	1757	1762	20,982	0.02	18,151	0.02	1.571	ns
5	Linalool	93	17:31.0	00:01.4	1547	1544	15,457	0.01	14,273	0.02	0.674	ns
6	Geraniol	69	23:14.0	00:01.4	1844	1839	18,645	0.02	17,447	0.02	0.477	ns
7	*trans*-Farnesol	69	31:24.0	00:01.6	2350	2355	12,911	0.01	14,418	0.02	0.233	ns
	∑ Terpenic compounds						97,893	0.09	87,349	0.10	1.057	ns
8	3-Oxo-α-ionol	108	35:15.0	00:01.4	2641	-	27,266	0.03	31,116	0.04	0.921	ns
	∑ C_13_-norisoprenoids						27,266	0.03	31,116	0.04	1.808	ns
9	Ethyl 2-hydroxy-4-methylvalerate	69	17:31.0	00:01.3	1547	1547	188,279	0.18	74,936	0.09	33.650	*
10	Ethyl isopentyl succinate	101	24:17.0	00:01.7	1900	1897	52,727	0.05	20,056	0.02	23.051	*
11	Isoamyl lactate	45	18:06.0	00:01.3	1583	1583	136,300	0.13	75,713	0.09	22.459	*
12	Diethyl butanedioate	101	20:12.0	00:01.5	1686	1679	5,015,783	4.84	1,878,508	2.19	21.671	*
13	Decyl 2,2-dimethylpropanoate	70	23:14.0	00:01.3	1844	-	13,321	0.01	6138	0.01	14.324	*
14	Ethyl 3-hydroxypropionate	73	18:34.0	00:01.1	1597	-	185,916	0.18	83,103	0.10	14.318	*
15	Ethyl 3-formylpropionate	85	28:01.0	00:01.2	2145	-	24,799	0.02	13,960	0.02	13.896	*
16	Diethyl 2-hydroxypentanedioate	85	28:36.0	00:01.3	2143	-	418,513	0.40	209,095	0.24	12.786	*
17	Ethyl 2-phenylethyl oxalate	104	31:03.0	00:01.4	2337	-	7245	0.01	2795	0.00	12.337	*
18	Diethyl 2-methylbutanedioate	115	31:17.0	00:01.1	2346	-	6585	0.01	3881	0.00	12.317	*
19	Ethyl hydrogen succinate	128	31:45.0	00:01.1	2363	2368	6,682,871	6.44	5,000,549	5.84	11.107	*
20	Ethyl pyruvate	43	11:27.0	00:01.2	1268	1267	114,024	0.11	181,394	0.21	9.854	*
21	Ethyl 2-acetamido-4-methylpentanoate	128	27:47.0	00:01.4	2117	-	7613	0.01	3598	0.00	9.168	*
22	Methyl 4-hydroxybutanoate	74	21:50.0	00:01.1	1757	-	7964	0.01	16,086	0.02	8.420	*
23	Diethyl malate	117	26:37.0	00:01.3	2031	2041	211,154	0.20	128,189	0.15	7.498	*
24	Methyl ethyl succinate	115	19:23.0	00:01.4	1641	1632	23,799	0.02	10,165	0.01	7.464	*
25	Ethyl 2-hydroxypropanoate	45	13:05.0	00:01.1	1344	1353	2,411,977	2.33	1,963,777	2.29	5.328	*
26	2-Phenylethyl propionate	104	24:03.0	00:01.7	1892	-	7125	0.01	5276	0.01	4.853	*
27	Methyl 2-methyl-3-oxobutanoate	88	26:02.0	00:01.2	2000	-	7922	0.01	10,553	0.01	4.573	*
28	Ethyl linoleate	105	34:47.0	00:02.2	2517	-	38,773	0.04	22,431	0.03	4.163	ns
29	Ethyl laurate	88	23:21.0	00:02.2	1844	1846	127,797	0.12	27,741	0.03	3.102	ns
30	Ethyl 2-phenylacetate	91	22:18.0	00:01.6	1793	1786	83,794	0.08	65,586	0.08	2.936	ns
31	Ethyl octanoate	88	15:11.0	00:02.0	1440	1440	1,534,067	1.48	1,216,408	1.42	2.934	ns
32	Ethyl heptanoate	88	12:58.0	00:01.9	1322	1327	8473	0.01	11,930	0.01	2.919	ns
33	α-Terpinyl acetate	59	20:33.0	00:01.5	1696	1693	13,056	0.01	10,422	0.01	2.915	ns
34	Ethyl undecenoate	152	38:24.0	00:01.2	2883	-	8752	0.01	4496	0.01	2.913	ns
35	Ethyl pentadecanoate	88	30:00.0	00:02.3	2268	2161	75,987	0.07	41,633	0.05	2.587	ns
36	Ethyl vanillate	151	35:15.0	00:01.3	2641	2653	4552	0.00	7188	0.01	2.452	ns
37	Ethyl dec-9-enoate	88	20:26.0	00:02.0	1693	1703	67,674	0.07	42,785	0.05	2.026	ns
38	Ethyl decanoate	88	19:30.0	00:02.1	1645	1642	330,113	0.32	201,810	0.24	1.944	ns
39	Ethyl 4-hydroxybutanoate	87	22:32.0	00:01.2	1800	1796	6,613,274	6.38	7,833,802	9.14	1.694	ns
40	Ethyl acetaminoacetate	72	28:22.0	00:01.2	2155	-	25,266	0.02	21,895	0.03	1.583	ns
41	Ethyl 3-cyclohexylpropanoate	88	31:10.0	00:01.4	2341	-	7943	0.01	6381	0.01	1.527	ns
42	Ethyl 3-hydroxyoctanoate	117	24:03.0	00:01.4	1892	1892	29,452	0.03	24,961	0.03	1.226	ns
43	Methyl 2,3-dihydroxybenzoate	136	30:00.0	00:01.2	2268	-	11,774	0.01	6125	0.01	1.179	ns
44	Ethyl 2-(4-hydroxyphenyl)acetate	180	38:52.0	00:01.2	2904	-	6564	0.01	7742	0.01	0.953	ns
45	Ethyl 3-hydroxybutanoate	71	16:56.0	00:01.2	1510	1505	53,677	0.05	44,603	0.05	0.890	ns
46	Ethyl 3-hydroxyhexanoate	71	20:12.0	00:01.3	1686	1690	9462	0.01	8390	0.01	0.559	ns
47	N-Acetyl-L-valine ethyl ester	72	26:23.0	00:01.4	2019	-	17,871	0.02	14,638	0.02	0.366	ns
48	Methyl pyruvate	43	21:57.0	00:01.4	1761	1217	122,105	0.12	137,910	0.16	0.199	ns
49	Ethyl hexanoate	88	10:38.0	00:01.8	1232	1238	810,509	0.78	862,228	1.01	0.155	ns
50	Ethyl 4-acetoxybutanoate	87	21:01.0	00:01.5	1732	-	30,519	0.03	32,622	0.04	0.110	ns
51	2-Phenylethyl acetate	104	22:53.0	00:01.6	1811	1811	1,474,492	1.42	1,411,052	1.65	0.084	ns
52	2-Methylbutyl acetate	43	08:04.0	00:01.6	1131	1128	3,851,619	3.71	4,093,266	4.78	0.072	ns
53	Ethyl 4-hydroxybenzoate	121	40:09.0	00:01.2	2996	-	13,799	0.01	14,950	0.02	0.060	ns
54	Ethyl 2-phenylethyl dimethylmalonate	104	37:35.0	00:01.4	2811	-	12,272	0.01	11,503	0.01	0.059	ns
55	Diisoproply phthalate	149	36:04.0	00:01.8	2702	-	7430	0.01	7370	0.01	0.001	ns
56	Hexyl acetate	43	11:34.0	00:01.7	1270	1275	46,249	0.04	45,864	0.05	0.000	ns
	∑ Esters						30,961,232	29.85	25,925,505	30.26	6.558	*
57	2,4,7,9-Tetramethyl-5-decyne-4,7-diol	109	27:26.0	00:01.3	2106	-	2419	0.00	4759	0.01	16.214	*
58	3-Methylpentan-1-ol	56	12:44.0	00:01.1	1316	1340	1,029,814	0.99	510,705	0.60	8.382	*
59	2,7-Dimethyloctane-4,5-diol	69	21:22.0	00:01.1	1743	-	56,054	0.05	37,359	0.04	7.861	*
60	2-(4-Methoxyphenyl) ethanol	121	31:03.0	00:01.3	2337	2335	54,485	0.05	41,628	0.05	7.723	*
61	2-Phenylethanol	45	24:24.0	00:01.2	1904	1909	9,329,193	8.99	4,663,562	5.44	7.509	*
62	Nonan-2-ol	45	16:56.0	00:01.4	1510	1528	60,114	0.06	44,710	0.05	7.000	*
63	4-Methylpentan-1-ol	56	12:23.0	00:01.1	1306	1301	138,557	0.13	57,418	0.07	5.757	*
64	4-Hexen-3-ol	71	28:08.0	00:01.2	2148	-	31,532	0.03	27,392	0.03	4.185	ns
65	*cis*-4-Hydroxymethyl-2-methyl-1,3-dioxolane	103	20:05.0	00:01.1	1682	-	40,436	0.04	112,028	0.13	3.949	ns
66	3-heptyn-2-ol	43	24:10.0	00:01.1	1896	-	140,268	0.14	193,684	0.23	3.516	ns
67	3-Ethyl-4-methyl-1-pentanol	69	16:42.0	00:01.3	1503	1507	3100	0.00	15,929	0.02	2.985	ns
68	*trans*-4-hydroxymethyl-2-methyl-1,3-dioxolane	103	18:55.0	00:01.1	1607		35,623	0.03	116,338	0.14	2.303	ns
69	3-Hexen-1-ol	67	14:01.0	00:01.2	1386	1380	173,450	0.17	117,245	0.14	2.180	ns
70	3-Ethoxy-1-propanol	59	13:47.0	00:01.1	1363	1377	44,867	0.04	24,178	0.03	2.092	ns
71	2,6-Dimethyl-7-octen-2,6-diol	71	25:27.0	00:01.2	1959	1964	7915	0.01	7044	0.01	1.734	ns
72	Heptan-1-ol	70	15:39.0	00:01.3	1453	1456	2,585,574	2.49	2,431,741	2.84	1.035	ns
73	Phenoxyethanol	94	28:15.0	00:01.2	2151	2142	10,304	0.01	12,013	0.01	0.683	ns
74	1-Butanol	56	08:25.0	00:01.1	1140	1146	140,783	0.14	154,607	0.18	0.429	ns
75	Isoamyl alcohol	55	10:10.0	00:04.9	1221	1230	445,386	0.43	395,568	0.46	0.357	ns
76	Pentan-1-ol	42	10:59.0	00:01.1	1241	1244	43,391	0.04	37,469	0.04	0.219	ns
77	2-Methyl-3-butene-1,2-diol	71	23:49.0	00:02.2	1863	-	17,499	0.02	16,048	0.02	0.175	ns
78	Butane-1,3-diol	45	18:06.0	00:01.0	1583	1576	1,564,242	1.51	1,473,432	1.72	0.109	ns
79	(2S,3S)-Butane-2,3-diol	45	17:24.0	00:01.0	1543	1545	5,140,609	4.96	5,339,535	6.23	0.084	ns
80	(3,4,5-Trimethoxyphenyl) methanol	198	38:31.0	00:01.4	2889	-	9875	0.01	10,396	0.01	0.020	ns
81	4-Methyl-5-thiazoleethanol	113	30:42.0	00:01.2	2300	2311	17,739	0.02	18,441	0.02	0.019	ns
	∑ Alcohols						21,123,230	20.37	15,863,228	18.52	7.556	*
82	Hexanoic acid	60	23:21.0	00:01.1	1848	1854	7,588,282	7.32	5,184,808	6.05	10.652	*
83	2-Oxopentanedioic acid	101	37:35.0	00:01.1	2811	-	177,608	0.17	121,733	0.14	9.957	*
84	Dodecanoic acid	60	33:16.0	00:01.2	2489	-	62,061	0.06	24,576	0.03	9.084	*
85	Isovaleric acid	60	20:05.0	00:01.0	1682	1680	7,103,710	6.85	5,799,112	6.77	8.570	*
86	Butanoic acid	60	19:16.0	00:01.0	1638	1637	1,073,005	1.03	828,020	0.97	7.530	*
87	Acetic acid	60	15:39.0	00:01.0	1453	1465	499,185	0.48	741,473	0.87	7.121	*
88	Caprylic acid	60	26:58.0	00:01.1	2050	2046	6,104,658	5.89	4,619,455	5.39	6.266	*
89	Octanoic acid	60	27:12.0	00:01.1	2098	2096	6,104,658	5.89	4,619,455	5.39	6.266	*
90	Succinic acid	56	30:49.0	00:01.0	2304	-	375,686	0.36	211,649	0.25	4.411	*
91	Butanedioic acid	56	37:00.0	00:00.9	2782	-	308,343	0.30	147,377	0.17	4.215	ns
92	Dec-9-enoic acid	69	31:10.0	00:01.1	2341	2341	279,052	0.27	184,202	0.22	3.734	ns
93	Decanoic acid	60	30:14.0	00:01.2	2279	2275	1,334,398	1.29	965,662	1.13	3.674	ns
94	4-Methyl-2-oxovaleric acid	57	15:25.0	00:01.3	1447	-	66,486	0.06	19,862	0.02	3.495	ns
95	5-Oxotetrahydrofuran-2-carboxylic acid	103	38:31.0	00:01.1	2889	-	80,224	0.08	60,831	0.07	3.393	ns
96	2-Methylbutanoic acid	74	20:05.0	00:01.1	1682	1674	5,108,137	4.93	4,341,982	5.07	2.652	ns
97	3-Hexenoic acid	68	25:13.0	00:01.0	1952	-	6614	0.01	3529	0.00	2.602	ns
98	Malic acid	71	37:28.0	00:01.0	2806		277,579	0.27	215,559	0.25	2.528	ns
99	2-Hydroxy-4-methylpentanoic acid	76	33:44.0	00:01.0	2592	-	75,528	0.07	37,324	0.04	1.723	ns
100	Hexadecanoic acid	60	38:45.0	00:01.4	2900	2900	77,788	0.07	60,127	0.07	1.506	ns
101	5-Hexenoic acid	60	24:24.0	00:01.0	1904	1900	53,357	0.05	34,362	0.04	0.869	ns
102	4-Methoxy-4-oxobutanoic acid	101	31:17.0	00:01.0	2346	-	40,500	0.04	33,313	0.04	0.776	ns
103	o-Anisic acid	105	37:07.0	00:01.2	2792	-	8878	0.01	6783	0.01	0.748	ns
104	Heptanoic acid	60	25:13.0	00:01.1	1952	1960	46,171	0.04	52,421	0.06	0.716	ns
105	Homovanillic acid	137	40:02.0	00:01.4	2992	3099	6456	0.01	5866	0.01	0.648	ns
106	Pyruvic acid	85	32:20.0	00:01.0	2411	-	7576	0.01	6720	0.01	0.574	ns
107	*trans*-3-Hexenoic acid	68	24:59.0	00:01.0	1923	1915	4188	0.00	3508	0.00	0.445	ns
108	2-(4-Hexyl-2,5-dioxofuran-3-yl)acetic acid	126	27:40.0	00:01.6	2113	2110	23,221	0.02	16,851	0.02	0.437	ns
109	2-Propenoic acid	45	19:30.0	00:01.0	1645	-	22,808	0.02	33,948	0.04	0.414	ns
110	Benzeneacetic acid	91	34:19.0	00:01.1	2572	2565	489,308	0.47	537,374	0.63	0.368	ns
111	Propionic acid	74	17:24.0	00:01.0	1543	1547	58,564	0.06	56,625	0.07	0.137	ns
112	Benzoic acid	105	32:34.0	00:01.1	2423	2423	26,705	0.03	25,854	0.03	0.104	ns
113	Isobutyric acid	73	18:06.0	00:01.0	1583	1581	539,147	0.52	559,134	0.65	0.027	ns
114	*trans*-2-Hexenoic acid	73	25:27.0	00:01.0	1959	1969	9305	0.01	9517	0.01	0.027	ns
115	7-Octenoic acid	57	27:54.0	00:01.1	2120	-	14,525	0.01	14,436	0.02	0.010	ns
116	Pentanoic acid	60	21:22.0	00:01.0	1743	1744	76,463	0.07	77,245	0.09	0.008	ns
117	Nonanoic acid	60	28:36.0	00:01.1	2162	2165	12,275	0.01	12,228	0.01	0.001	ns
	∑ Acids						38,142,449	36.78	29,672,925	34.64	17.718	*
118	2-Ethoxy-6-(methoxymethyl)phenol	137	31:17.0	00:01.4	2346	-	6209	0.01	2609	0.00	46.882	*
119	4-Vinylguaiacol	135	29:11.0	00:01.3	2200	2203	287,108	0.28	483,388	0.56	3.766	ns
120	2,4-Ditert-butylphenol	191	30:49.0	00:01.3	2304	2316	8225	0.01	6636	0.01	1.416	ns
121	2,6-Ditert-butyl-4-methylphenol	205	24:31.0	00:02.0	1908	1906	85,342	0.08	91,286	0.11	1.015	ns
122	Phenol	94	26:09.0	00:01.1	2000	2008	15,548	0.01	16,802	0.02	0.864	ns
	∑ Phenols						402,432	0.39	600,722	0.70	3.701	ns
123	4-Hydroxybenzaldehyde	121	39:48.0	00:01.2	2960	2958	140,741	0.14	97,154	0.11	4.282	*
124	Benzaldehyde	106	17:10.0	00:01.4	1536	1534	6935	0.01	8158	0.01	3.959	ns
125	6,6-Trimethyl-1-cyclohexene-1-propenal	163	24:59.0	00:01.6	1923		3759	0.00	4395	0.01	1.300	ns
126	Benzeneacetaldehyde	91	19:37.0	00:01.4	1648	1648	52,327	0.05	56,856	0.07	0.159	ns
127	Hydroxy methyl furfural	97	33:30.0	00:01.1	2500	-	22,283	0.02	22,031	0.03	0.027	ns
	∑ Aldehydes						226,044	0.22	188,594	0.22	2.768	ns
128	2-Methyl-4-phenyl-3-pentanone	105	25:27.0	00:02.0	1959	-	18,102	0.02	104,172	0.12	8.945	*
129	3-Hydroxy-2-butanone	45	11:48.0	00:01.1	1276	1280	59,058	0.06	21,029	0.02	4.706	*
130	1-Phenylethanone	105	19:44.0	00:01.4	1652	1656	18,532	0.02	21,882	0.03	3.013	ns
131	4,5-Dimethyl-1,3-dioxol-2-one	114	27:54.0	00:01.1	2120	-	22,813	0.02	24,528	0.03	0.452	ns
132	Acetovanillone	151	35:29.0	00:01.2	2650	2651	93,840	0.09	85,845	0.10	0.162	ns
133	Zingerone	137	37:21.0	00:01.3	2800	2790	7157	0.01	6754	0.01	0.083	ns
	∑ Ketones						219,503	0.21	264,211	0.31	0.484	ns
134	2-Benzofuran-1(3H)-one	105	31:38.0	00:01.4	2359	2356	4335	0.00	6958	0.01	18.421	*
135	δ-Valerolactone	42	22:46.0	00:01.3	1808	-	28,596	0.03	63,074	0.07	11.676	*
136	DL Mevalolactone	71	34:12.0	00:01.1	2566	-	36,733	0.04	54,671	0.06	10.112	*
137	2,3-Dihydro-1-benzofuran	120	31:59.0	00:01.1	2371	2389	574,035	0.55	1,642,628	1.92	7.665	*
138	5-(Hydroxymethyl)dihydrofuran-2(3H)-one	85	35:50.0	00:01.1	2664	-	3,008,921	2.90	2,223,477	2.60	5.411	*
139	δ-Octalactone	99	25:34.0	00:01.5	1963	1965	29,764	0.03	21,141	0.02	4.794	*
140	3-Hydroxy-4,4-dimethyldihydrofuran-2(3H)-one	128	26:30.0	00:01.1	2025	-	2356	0.00	2954	0.00	4.547	*
141	4-Hydroxy-2-ethyl-5-methyl-3(2H)-furanone	56	27:26.0	00:01.1	2106		22,976	0.02	16,587	0.02	4.410	*
142	4-(1-Hydroxyethyl)-γ-butanolactone	86	31:03.0	00:01.2	2337	2328	51,285	0.05	39,634	0.05	2.961	ns
143	5-Ethoxydihydro-2(3H)-furanone	85	21:08.0	00:01.4	1735	1728	96,235	0.09	67,581	0.08	2.558	ns
144	δ-Hexanolactone	42	22:25.0	00:01.4	1796	1792	53,111	0.05	28,094	0.03	2.431	ns
145	α-Amino-γ-butyrolactone	57	28:43.0	00:01.1	2165	-	15,428	0.01	17,230	0.02	2.156	ns
146	*cis*-4-Hydroxy-3-methylundecanoic acid lactone	99	25:41.0	00:01.6	1967	-	22,731	0.02	17,913	0.02	1.168	ns
147	γ-Hexalactone	85	20:47.0	00:01.4	1704	1703	12,815	0.01	14,975	0.02	0.815	ns
148	3-Hydroxy-4,4-dimethyldihydrofuran-2(3H)-one	71	26:37.0	00:01.1	2031	2034	108,610	0.10	115,306	0.13	0.672	ns
149	δ-Decalactone	99	29:11.0	00:01.6	2200	2192	26,224	0.03	24,117	0.03	0.420	ns
150	5-(Hydroxy[methoxy(5-oxotetrahydro-2-furanyl)methoxy]methyl)dihydro-2(3H)-furanone	85	31:24.0	00:01.2	2360	-	30,839	0.03	25,851	0.03	0.411	ns
151	γ-Octalactone	85	24:38.0	00:01.5	1911	1916	40,055	0.04	46,792	0.05	0.210	ns
152	γ-Nonalactone	85	28:22.0	00:01.6	2155		7610	0.01	7212	0.01	0.165	ns
153	3,4-Dihydroxy-5-methyl-dihydrofuran-2-one	60	38:59.0	00:01.1	2908	-	20,306	0.02	21,262	0.02	0.056	ns
154	Ƴ-Butyrolactone	68	22:32.0	00:01.1	1800	-	3,075,844	2.97	3,137,551	3.66	0.014	ns
155	γ-Heptalactone	85	22:39.0	00:01.4	1804	1796	9714	0.01	9998	0.01	0.012	ns
	∑ Lactones and Furanoids						7,278,522	7.02	7,605,007	8.88	0.347	ns
156	Ethyl 3-methylthiopropanoate	74	18:06.0	00:01.5	1583	1580	15,294	0.01	38,729	0.05	6.358	*
157	3-(Methylthio)propionic acid	61	30:35.0	00:01.0	2295	2298	119,593	0.12	72,314	0.08	6.004	*
158	2-Methyldihydrothiophen-3(2H)-one	60	17:17.0	00:01.5	1540	-	822,873	0.79	550,872	0.64	3.146	ns
159	3-(Ethylthio)propanol	61	22:04.0	00:01.2	1785	1802	30,717	0.03	40,517	0.05	2.897	ns
160	N-acetylmethionine ethyl ester	99	35:50.0	00:01.4	2664	-	14,010	0.01	15,774	0.02	0.919	ns
161	3-Methylthiopropyl acetate	61	19:16.0	00:01.5	1638	1633	6213	0.01	7236	0.01	0.669	ns
162	S-(3-Hydroxypropyl) ethanethioate	74	25:48.0	00:01.2	1971	-	76,117	0.07	86,477	0.10	0.660	ns
163	5-Acetyldihydrofuran-2(3H)-one	85	27:05.0	00:01.2	2092	2096	23,593	0.02	22,169	0.03	0.388	ns
164	3-Methylmercapto-1-propanol	106	20:54.0	00:01.2	1707	1715	1,677,408	1.62	1,720,142	2.01	0.035	ns
	∑ Sulfur-containing compounds						2,785,819	2.69	2,554,230	2.98	0.601	ns
165	2-Ethylbutan-1-amine	101	35:08.0	00:01.1	2636	-	192,419	0.19	98,372	0.11	26.914	*
166	N-Phenethylacetamide	104	34:33.0	00:01.3	2583	2590	10,957	0.01	72,921	0.09	8.741	*
167	3-Methylpiperazine-2,5-dione	85	34:05.0	00:01.0	2560	-	5863	0.01	4539	0.01	5.000	ns
168	Benzothiazole	135	25:20.0	00:01.5	1956	1959	8013	0.01	4187	0.00	3.423	ns
169	N,N-Dibutylformamide	72	21:57.0	00:01.7	1761	1767	9573	0.01	12,233	0.01	1.776	ns
170	N-Acetylcysteamine	60	15:18.0	00:01.3	1443	-	16,226	0.02	30,705	0.04	1.708	ns
171	1H-indole	117	32:48.0	00:01.2	2434	2435	9900	0.01	34,798	0.04	1.101	ns
172	N-(3-Methylbutyl)acetamide	72	23:35.0	00:01.2	1856	1855	3509	0.00	7045	0.01	1.042	ns
173	1H-Isoindole-1,3(2H)-dione	120	39:13.0	00:01.3	2940	-	137,672	0.13	122,523	0.14	0.982	ns
174	3-Ethyl-4-methyl-1H-pyrrole-2,5-dione	139	30:14.0	00:01.2	2279	2260	20,059	0.02	22,456	0.03	0.453	ns
175	2-(Oxan-4-yl)ethanamine	85	28:22.0	00:01.1	2155	-	10,950	0.01	10,492	0.01	0.138	ns
176	2-Propoxyethylamine	68	36:39.0	00:00.9	2730	-	3229	0.00	3729	0.00	0.037	ns
	∑ Nitrogen-containing compounds						428,370	0.41	424,000	0.49	0.003	ns
177	4-Hydroxy-6-pentyltetrahydro-2H-pyran-2-one	102	37:28.0	00:01.5	2806	-	6418	0.01	2634	0.00	14.628	*
178	1,1-Di(2-methyl butoxy)ethane	71	12:16.0	00:02.5	1303	-	169,495	0.16	688,162	0.80	7.905	*
179	Succinic acid anhydride	56	35:01.0	00:01.0	2631	-	450,941	0.43	285,403	0.33	7.558	*
180	2-Methyl-2-propyl-1,3-dioxolane	87	35:43.0	00:01.3	2659	-	4732	0.00	6170	0.01	1.151	ns
181	Isothiocyanatocyclohexane	141	20:12.0	00:02.0	1686	1670	17,086	0.02	17,710	0.02	1.141	ns
182	1,4-Dioxanyl hydroperoxide	115	20:05.0	00:01.7	1682	-	7069	0.01	9086	0.01	0.946	ns
183	2,3-Diphenylbutane	105	18:55.0	00:01.6	1607	-	3817	0.00	4115	0.00	0.157	ns
184	2-Methyl-2H-pyran-3,4,5 (6H)-trione	142	30:35.0	00:01.1	2296	-	2767	0.00	2992	0.00	0.137	ns
185	Methylsuccinic anhydride	68	23:07.0	00:01.1	1841	1855	4237	0.00	5186	0.01	0.086	ns
186	4,5-Dimethyl-2-pentadecyl-1,3-dioxolane	101	33:37.0	00:01.1	2500		52,080	0.05	50,362	0.06	0.045	ns
187	Ethoxy-1-pentoxyethane	73	07:43.0	00:02.0	1103	1104	1,298,503	1.25	1,373,564	1.60	0.043	ns
188	*trans*-7-tetradecene	83	15:25.0	00:02.8	1447	1435	8217	0.01	8055	0.01	0.034	ns
	∑ Other compounds						2,025,363	1.95	2,453,438	2.86	0.775	ns

^1^ t_R_—retention time in first chromatographic dimensions; ^2^ t_R_—retention time in second chromatographic dimensions; LRI_lit_—linear retention index from the literature; LRI_exp_—linear retention index obtained experimentally; S—statistical differences; -—not found; ns—no significant differences; and *—significant differences (*p* < 0.05). *Cis* and *trans* indicate geometric isomers and are written in italic type.

## Data Availability

All data are contained within the article and supplementary materials.

## Supplementary Materials

The following supporting information can be downloaded at: www.mdpi.com/article/10.3390/metabo12121295/s1, Table S1: General vineyard parameters; Table S2: Climate data obtained from the Croatian Meteorological and Hydrological Service in the period from January 2021 to September 2021 and represented the average value of four vineyards for the Northern Dalmatia subregion and six vineyards for the Central and Southern Dalmatia subregions; Table S3: Odor activity value (OAV) of the main odorants in young Maraština wines quantified by gas chromatography–mass spectrometry (GC-MS/MS) in Central and Southern Dalmatia and Northern Dalmatia subregions.
