# Supplementary material for: A Targeted and an Untargeted Metabolomics Approach to the Volatile Aroma Profile of Young ‘Maraština’ Wines
Source: Metabolites. 2022 Dec 19;12(12):1295. doi: 10.3390/metabo12121295 (PMC9780986; doi:10.3390/metabo12121295)
Supplement: Supplementary file 1 [file metabolites-12-01295-s001.zip › Table S1.pdf]

Table S1. General vineyard parameters.

Vineyard	Geographical position	Altitude (m)	Vine-growing subregion	Meteorological station	Plantation year	Soil type	Row distance x vine, m	Trellis system	Row orientation
**Institute for Adriatic Crops and Karst Reclamation, Split**	43°30' 35'' N, 16°29' 85'' E	14	Central and Southern Dalmatia	Split	2005	brown	2.0 x 1.0	bilateral cordon	north-south
**Dračevica (Korčula)**	42°55' 36'' N, 16° 54' 0'' E	62	Central and Southern Dalmatia	Vela Luka (Korčula)	1941	brown	1.5 x 1.2	bilateral cordon	north-south
**Prapatna 1 (Korčula)**	42°54' 51'' N, 16°54' 58'' E	40	Central and Southern Dalmatia	Vela Luka (Korčula)	2000	brown-redish	1.8 x 1.0	bilateral cordon	north.south
**Prapatna 2 (Korčula)**	42° 54' 50'' N, 16° 54' 52'' E	40	Central and Southern Dalmatia	Vela Luka (Korčula)	2008	brown	1.2 x 1.0	bilateral cordon	north-south
**Kruševo (Korčula)**	42° 55' 20'' N, 16° 53' 54'' E	68	Central and Southern Dalmatia	Vela Luka (Korčula)	1999	brown-redish	1.8 x 1.0	bilateral cordon	north-south
**Kaštela (Kaštel Kambelovac)**	43° 33' 34'' N, 16° 22' 32'' E	94	Central and Southern Dalmatia	Split	2011	brown-redish	1.6 x 0.9	bilateral cordon	north-south
**Smilčić**	44° 7' 23'' N, 15° 28' 52'' E	60	Northern Dalmatia	Zadar	2009	brown	2.6 x 1.0	bilateral cordon	north-south
**Nadin (Polača)**	44° 0' 28'' N, 15° 29' 41'' E	103	Northern Dalmatia	Benkovac	2010	reclaime d karst	2.2 x 1.1	bilateral cordon	north-south
**Stankovci**	43° 57' 20'' N, 15° 43' 6'' E	130	Northern Dalmatia	Benkovac	2008	brown	1.9 x 1.0	bilateral cordon	north-south
**Vukšić**	43° 56' 37'' N, 15° 43' 46'' E	130	Northern Dalmatia	Benkovac	2003	brown	2.0 x 1.0	bilateral cordon	north-south
