# Supplementary material for: A Targeted and an Untargeted Metabolomics Approach to the Volatile Aroma Profile of Young ‘Maraština’ Wines
Source: Metabolites. 2022 Dec 19;12(12):1295. doi: 10.3390/metabo12121295 (PMC9780986; doi:10.3390/metabo12121295)
Supplement: Supplementary file 1 [file metabolites-12-01295-s001.zip › Table S2.pdf]

Table S2: Climate data obtained from the Croatian Meteorological and Hydrological Service in the period from January 2021 to September 2021 and represented the average value of four vineyards for the Northern Dalmatia subregion and six vineyards for the Central and Southern Dalmatia subregion.

Vine-growing subregion	Month	Tav (˚C)	Tmin (˚C)	Tmax (˚C)	Mp (mm)	Ms (h)
Northern Dalmatia	January	7	3	10	145	95
	February	9	5	13	45	126
	March	10	5	15	15	249
	April	12	7	16	50	250
	May	17	12	22	19	291
	June	24	18	29	3	365
	July	26	20	31	40	334
	August	25	19	30	29	333
	September	22	16	27	51	281
Central and Southern Dalmatia	January	8	5	11	128	92
	February	10	6	14	50	152
	March	10	6	14	29	238
	April	13	8	17	42	228
	May	19	14	23	66	283
	June	25	19	30	5	347
	July	28	22	33	10	349
	August	27	22	32	94	342
	September	22	17	27	24	290

Tav- average temperature, Tmin-average minimum temperature, Tmax-average maximum temperature, Mp-average precipitation, Ms-average hours of sunshine.
