# Supplementary material for: A Targeted and an Untargeted Metabolomics Approach to the Volatile Aroma Profile of Young ‘Maraština’ Wines
Source: Metabolites. 2022 Dec 19;12(12):1295. doi: 10.3390/metabo12121295 (PMC9780986; doi:10.3390/metabo12121295)
Supplement: Supplementary file 1 [file metabolites-12-01295-s001.zip › Table S3.pdf]

Table S3. Odor activity value (OAV) of the main odorants in young Maraština wines quantified by gas chromatography-mass spectrometry (GC-MS/MS) in Central and Southern Dalmatia and Northern Dalmatia subregions.

No.	Volatile compound	Concentration (μg/L)		ODT (μg/L)*	Odor descriptor*	OAV	
		ND	CSD			ND	CSD
1	*β*-Damascenone	1.89	0.84	0.05	honey,prunes	38	17
2	Ethyl caprylate	221.09	176.73	5	fruity, sweet	44	35
3	Ethyl isovalerate	11.06	8.02	3	fruity, lemon	4	3
4	Ethyl 2-methylbutyrate	7.55	5.39	1	apple, strawberry	7	5
5	Ethyl caproate	178.75	161.34	14	green,apple,fruity	13	11
6	Isopentyl acetate	491.93	579.53	30	banana	16	19
7	Ethyl butyrate	67.10	66.89	20	buttery	3	3
8	4-Vinylguaiacol	155.17	271.54	40	spicy, clove, curry	4	7
9	Phenylacetaldehyde	39.96	31.85	4	honey	10	8
10	Octanoic acid	2453.81	2083.90	500	fatty, cheese, rancid	5	4

ODT-odor thresholds; ND- Northern Dalmatia subregion; CSD- Central and Southern Dalmatia subregion. The OAV calculates as quotients of concentration of a specific compound and the corre-sponding odor detection threshold from the literature

*Odor thresholds (μg/L) and odor descriptors reported in the literature [32,34,36,41,43,53].
